# Treatment of Childhood Obesity Based on Brazilian Dietary Guidelines Plus Energy Restriction (PAPPAS HUPE Study): Protocol for a Randomized Clinical Trial

**DOI:** 10.2196/16170

**Published:** 2020-06-08

**Authors:** Joana Maia Brandao, Rosely Sichieri, Simone Augusta Ribas, Eliseu Verly-Jr, Rosangela Alves Pereira, Inês Rugani Ribeiro De Castro, Bruna Kulik Hassan, Alessandra Silva Dias De Oliveira, Emanuele Souza Marques, Diana Barbosa Cunha

**Affiliations:** 1 Department of Epidemiology Social Medicine Institute State University of Rio de Janeiro Rio de Janeiro Brazil; 2 Department of Nutrition and Public Health School of Nutrition Federal University of Rio de Janeiro Rio de Janeiro Brazil; 3 Department of Social and Applied Nutrition Federal University of Rio de Janeiro Rio de Janeiro Brazil; 4 Department of Social Nutrition Nutrition Institute State University of Rio de Janeiro Rio de Janeiro Brazil

**Keywords:** pediatric obesity, clinical trial, food guide

## Abstract

**Background:**

The Food Guide for the Brazilian Population relies on natural or minimally processed foods mainly of plant origin such as beans and rice with low oil, salt, and sugar content and limited consumption of ultraprocessed foods. Reduction of ultraprocessed foods improves diet quality and energy consumption.

**Objective:**

The goal of this study is to evaluate the effectiveness of an intervention for the treatment of obesity in children, with counseling based on the Brazilian Food Guide plus control of total energy intake.

**Methods:**

A parallel, randomized clinical trial will include children aged 7 to 12 years. Randomization will be performed in blocks of 10 individuals using computer-generated random sequence numbers. Both the control and intervention groups will participate in 6 standardized educational activities based on the 10 steps of the Brazilian Food Guide. These activities will be conducted at the University Hospital Toy Library, located in the pediatric outpatient clinic. For the intervention group, in addition to the educational activities, an individualized food plan based on the nutritional recommendations of the Brazilian Society of Pediatrics will be prescribed and discussed with the mothers and fathers. The primary outcome of the study will be variations in body mass index, and secondary outcomes will include analysis of insulin resistance, blood pressure, body fat percentage, and waist and neck circumference.

**Results:**

This project was funded by the National Council for Scientific and Technological Development in December 2017 (grant no 408333/2017-0). Recruitment began in August 2018 and by September 2019, we had enrolled the 101 participants. In addition to the patients referred by the national system of regulation, recruitment was made by medical outpatient referral and external indication. This is an ongoing study. We expect the results to be published in November 2020.

**Conclusions:**

At the end of the project, in case of a positive result, a protocol for the treatment of obesity based on the Brazilian Food Guide will be proposed to the Unified Health System. A successful method to reduce childhood obesity is expected.

**Trial Registration:**

Brazilian Registry of Clinical Trials RBR-3st5sn; http://www.ensaiosclinicos.gov.br/rg/RBR-3st5sn

**International Registered Report Identifier (IRRID):**

DERR1-10.2196/16170

## Introduction

The global prevalence of excessive weight among children aged 5 years and younger, which was 42 million in 2013, is expected to increase to 70 million in 2025 [1,2]. In Brazil, the increase in the prevalence of obesity over the past few decades has been higher among children aged 5 to 9 years compared with adolescents and adults, with an increase of approximately 6 times in the period from 1974 to 2009 [3]. During this period, Brazilian adolescents’ diets were low in vegetables and fruits with a high intake of sodium-rich food, sweets, and soft drinks [4-6].

Obesity before the onset of puberty increases the risk of adult type 2 diabetes, particularly if it continues until puberty or even later [7], indicating a window of opportunity to reduce obesity and related diseases later in life.

The World Health Organization (WHO) recommends interventions to control obesity in childhood. Accordingly, in 2014, the World Health Assembly adopted the Global Action Plan for the Prevention and Control of Noncommunicable Disease 2013-2020, which includes reducing global obesity rates among children, adolescents, and adults [8].

The 2014 Food Guide for the Brazilian Population [9] is a strategy for the implementation of the Adequate and Healthy Food promotion guideline that integrates the National Food and Nutrition Policy. The guide is intended for the population aged 2 years and older and classifies foods based on the degree of industrial processing. This classification, called the NOVA food classification system, comprises four groups: (1) unprocessed or minimally processed foods, (2) culinary ingredients, (3) processed foods, and (4) ultraprocessed foods [10,11].

High consumption of ultraprocessed foods has been associated with obesity, diabetes, and cardiovascular disease in different age groups [12-16]. Ultraprocessed foods are more energy-dense and contain higher levels of total fat, saturated fat, sugar, and salt and lower levels of protein and dietary fiber in comparison with unprocessed or minimally processed foods. In addition, they stimulate excessive consumption because of hyperpalatability, large portion sizes, and easy consumption. Therefore, they can be consumed as snacks anytime, anywhere and are often marketed intensively and persuasively. In a randomized trial with individuals eating ad libitum, the poor quality of a diet rich in ultraprocessed foods was also associated with greater energy intake when compared with a diet very low in ultraprocessed foods [17].

Obesity prevention reviews based on the promotion of positive eating behaviors have not achieved the desired impact [18,19], and trials for the treatment of obesity in the primary care setting are effective only when they include caloric restriction and parental involvement, as shown in the available literature reviews [20]. Reduction of energy intake associated with consumption of ultraprocessed foods may be improved by combining the NOVA classification with a food plan. Accordingly, a review of interventions to increase fruit and vegetable consumption among school children found improved targeted dietary behaviors; however, there were no effects on adiposity [21], suggesting that for the treatment of obesity, the amount of food consumed is also an important aspect to be considered. Thus, the aim of this project is to compare a Food Guide for the Brazilian Population–based intervention incorporating the NOVA classification of food with and without energy intake counseling. If effective, this proposal could guide the development of clinical protocols for primary care aimed at the treatment of obesity in children, a challenge in the Brazilian public health agenda.

## Methods

### Design and Study Population

Parents and Professionals for Healthy Eating–Pedro Ernesto University Hospital (PAPPAS HUPE) is a nonblind randomized clinical trial for the treatment of obesity in children referred by the National Regulatory System to the pediatric nutrition clinic of a university hospital located in the metropolitan region of the city of Rio de Janeiro, Brazil. Obese children aged 7 to 12 years are eligible to participate in the study. The exclusion criteria will be children diagnosed with genetic disorders associated with obesity (congenital leptin deficiency, Down syndrome, Prader-Willi syndrome) or endocrine disorder (hypothyroidism, Cushing syndrome) and patients already under nutritional monitoring or using weight loss medications. These data will be obtained through medical records and/or a questionnaire completed by parents.

**Figure 1:**
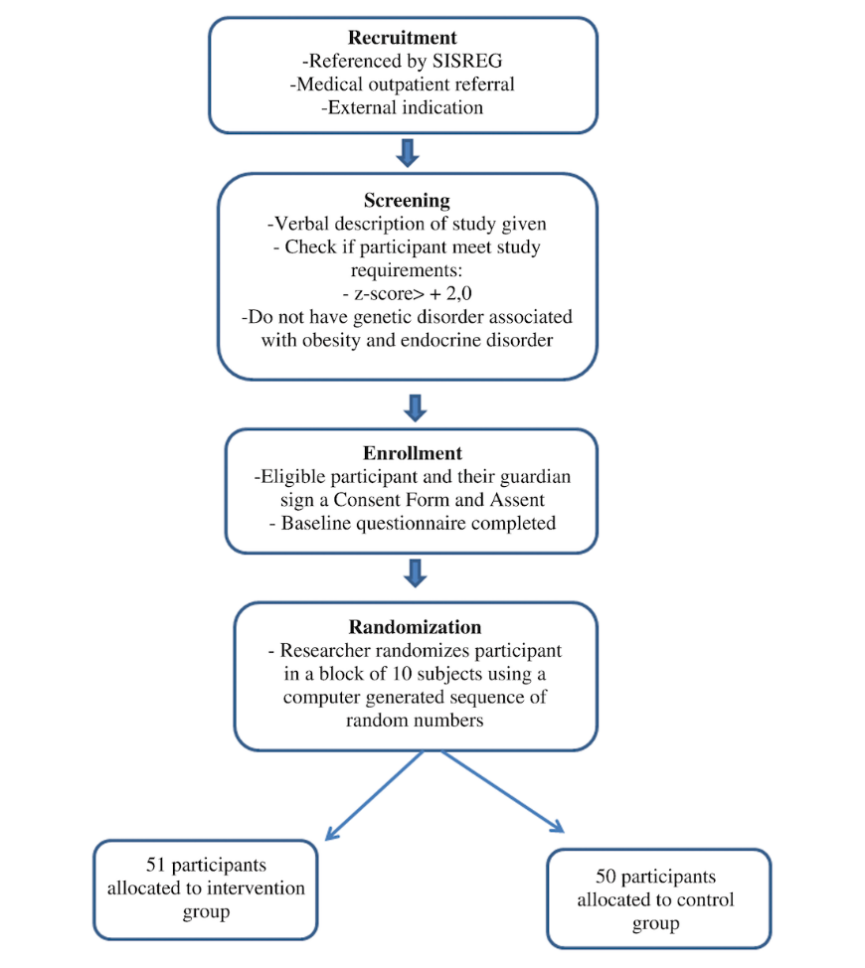
Consolidated Standards of Reporting Trials diagram. SISREG: Sistema Nacional de Regulação (National Regulation System).

The study will employ a parallel design with 2 comparison groups (Figure 1):

- Control group that will receive monthly nutritional guidance based on the new Food Guide for the Brazilian Population [9];
- Intervention group that will receive monthly nutritional guidance based on the new Food Guide for the Brazilian Population and a home-based diet plan appropriate to the nutritional needs of the participants.

The protocol will follow the guidelines of the Consolidated Standards of Reporting Trials [22] and was registered prospectively with the Brazilian Registry of Clinical Trials [RBR-3st5sn].

### Ethics Approval and Consent to Participate

The project was approved by the Research Ethics Committee of the Pedro Ernesto University Hospital (CAAE: 87593118000005259). The parent or legal guardian of the child will be informed of the need to sign a consent form and assent according to Brazilian Resolution number 466/2012 on research involving human beings from the Health Council of the Ministry of Health to authorize the information provided by the population in a study, emphasizing privacy regarding identification of the content as well as the freedom to withdraw from the research at any time.

### Sample Size Calculation and Randomization Procedure

The sample size of 48 individuals per group was calculated based on a standard deviation of body mass index (BMI) equal to 3.0 and an expected difference of 1.72 BMI [23] units between the groups, considering a statistical power of 80% and significance level of 5%. We estimate that 5% of participants are likely to drop out of the study during the study follow-up period. To account for this, we have enrolled 101 participants in total.

After the recruitment phase, eligible participants will be randomly assigned to the control or intervention groups. The randomization, which is performed by the dietitian responsible for the trial, will be performed in a block of 10 subjects using a computer-generated sequence of random numbers. The allocation implementation mechanism will be numbered sequentially. Participants will be allocated to each group, and interventions will be promoted by trained nutritionists.

### Intervention

Both groups will participate in 6 monthly standardized educational activities based on the 10 steps of the Food Guide for the Brazilian Population, which will be carried out in the Hospital Toy Library. Recreational materials, audiovisual resources, and pedagogical support necessary to carry out the proposed educational practice, already available, are based on the Activity Notebook–Promotion of Adequate and Healthy Food–Infant Education, developed by the Ministry of Health in partnership with the University of the State of Rio de Janeiro for teachers and health professionals (Table 1) [24]. Activities will be conducted in groups of 10 children. To avoid contamination, activities for the control and intervention groups will be conducted on alternate days.

**Table 1:** Description and objectives of the interventions.

Intervention	Description	Purpose of the activity
From food to meals	Addresses the following Food Guide steps: Step 1: make food in natura or consume minimally processed food Step 3: limit consumption of processed foods Step 4: avoid consumption of ultraprocessed foods	Enable participants to recognize food groups by degree of processing Facilitate reflection on the consumption of these food groups at home Help participants understand that usual meals should integrate fresh or minimally processed foods, enabling them to limit/avoid the consumption of processed and ultraprocessed foods
Fat, salt, and sugar	Addresses the following Food Guide step: Step 2: use oils, fats, salt, and sugar in small amounts by seasoning and cooking food	Make participants aware of the amount of fat, salt, and sugar present in food Help participants reflect on the amount of these ingredients in meals and on the possible consequences of high consumption
What a happy time	Addresses the following Food Guide steps: Step 5: eat with regularity and attention, in appropriate environments, and, whenever possible, with company Step 8: plan the use of time to give food the space it deserves	Help participants understand the concept and importance of commensality Help stimulate reflection about this practice
Going shopping	Addresses the following Food Guide step: Step 6: make purchases in places that offer varieties of food in natura or minimally processed foods	Help participants identify food sale locations close to where they live or study Make participants aware of the importance of shopping in places that offer varieties of food in natura or minimally processed foods Enable participants identify the characteristics that make places the most appropriate to buy their food
There is a child in the kitchen	Addresses the following Food Guide step: Step 7: develop, exercise, and share culinary skills	Encourage participants to engage in healthy meal preparation and develop and/or exercise their culinary skills Encourage participants to share the knowledge and recipes learned with family and friends
What do you have to eat and drink around here?	Addresses the following Food Guide steps: Step 9: give preference, when away from home, to places that serve meals made at the time and with food in natura or minimally processed foods Step 10: be critical about information, directions, and messages in commercial advertisements	Enable participants to make healthy choices when eating out Enable participants to critically analyze the advertisements and labels of food/products ready for consumption

Educational activities will also be held with parents addressing the same topics as those covered in the children’s workshops. Advice will include changes in family behavior related to food choices and purchases.

After the group activities, individual family consultations for the control and intervention groups will be held. In the control group, the counseling will reinforce lessons learned in educational activities, focusing on food quality. In the intervention group, a food plan will be prescribed for each participant based on nutritional recommendations of the Federal Council of Nutritionists and Brazilian Society of Pediatrics [25,26]. The plan will be reviewed monthly to match energy recommendations and weight change observed in the previous month.

In order to reduce follow-up losses and increase adherence to the study protocol, a telephone call in the week after the appointment will be made with those responsible for the children to evaluate potential difficulties in adhering to the guidelines provided. Those reporting difficulties in participating will be contacted weekly. This intervention description follows the template for the intervention description and replication checklist and guide [27].

### Data Collection

Those responsible for data collection will be trained to ensure a high quality of assessment. This training will be conducted with the objective of standardizing the measurement of data and ensuring reliability.

### Outcomes

The primary outcome of the study will be variations in BMI. The following secondary outcomes will be investigated: waist-to-height ratio (WHR), waist circumference (WC), neck circumference (NC), blood pressure, body fat percentage (BF%), and biochemical analysis of serum total cholesterol, low-density lipoprotein (LDL) cholesterol, high-density lipoprotein (HDL) cholesterol, triglycerides, glucose, and insulin resistance. Insulin resistance will be determined at the baseline and endpoint while the other biochemical analyses will be conducted at the baseline, midpoint, and endpoint. The remaining variables will be assessed at every consultation.

Body weight and BF% will be measured using a portable electronic scale (Tanita BC-558). Height will be checked in duplicate using an AlturaExata portable anthropometer (Belo Horizonte). Both measures will be collected with the children barefoot, wearing light clothes, with the arms extended from the side of the body, positioned by the Frankfurt horizontal plane [28,29]. To reduce potential BF% measurement errors, parents and children will be provided with guidelines regarding the intake of liquids, coffee, alcoholic beverages, laxatives, and diuretics prior to the anthropometric evaluation.

To measure WC and NC, a flexible and inelastic metric tape measuring 150 cm and a variation of 0.1 mm will be used. The WC will be measured with the tape placed horizontally at the midpoint between the lower edge of the last rib and the iliac crest [30]. The measurements will be performed with the tape firmly on the skin, without compression of the tissues. The evaluation will be made while standing with the abdomen relaxed and the arms extended out from the body. The WHR will be obtained by the quotient between WC and height, both in centimeters. The NC will be measured at the average neck height.

The classification of nutritional status will be based on BMI values for age, in z-scores, according to sex, based on the new curves proposed by WHO. BMI for age will be calculated using AnthroPlus 2007 software (WHO) [31], and the values obtained will be classified according to the recommended cutoff points for children aged 5 to 19 years: low weight (BMI for age <–2 z-scores), eutrophic (BMI for age ≥–2 and ≤+1 z-scores), overweight (BMI for age >+1 and ≤+2 z-scores), obese (BMI for age >+2 and <+3 z-scores), and severely obese (BMI for age >+3 z-scores). This classification will be performed at the baseline to confirm the inclusion criteria.

Blood pressure will be measured with the HEM-742 blood pressure monitor (Omron Healthcare Inc.), previously validated for use in adolescents [32]. Before taking the measure, it will be verified that the children do not have full bladders, have not taken medication and/or had coffee, and have not eaten up to 30 minutes before the procedure or engaged in physical exercise up to 1 hour before. The measurement will be performed on the right arm, at the level of the heart, supported on a table with a flat surface, with the palm facing up and the elbow slightly flexed. The individual should be seated with feet in contact with the floor or on a flat surface and remain at rest in that position for 5 minutes before the measurement [33]. Two measurements will be performed, with a minimum interval of 2 minutes, and the average of 2 measurements will be calculated. If the difference between the 2 measurements is equal to or greater than 5 mm Hg, a third measure will be taken.

Biochemical analysis of serum total cholesterol, LDL cholesterol, HDL cholesterol, triglycerides, and glucose will be determined at the baseline, midpoint, and endpoint. The biochemical analysis will be performed at the hospital’s central laboratory of clinical analyses. After 12 hours of fasting, 10 ml of blood from the left ventricle vein will be collected in vacutainer tubes. The samples will be divided into aliquots, placed in Eppendorf-type tubes, and stored at –80°C until use, when they will be processed and the serum analyzed in a biochemical analyzer (Cobas Integra 400 Plus/Cobas 6000, F Hoffman–La Roche Ltd) with a Roche cassette. Fasting glycemia will be determined by hexokinase, total cholesterol and triglycerides by the colorimetric method, and HDL cholesterol by the homogeneous method (F Hoffman–La Roche Ltd). LDL cholesterol will be calculated by the Friedewald et al [34] equation, recommended by the American Academy of Pediatrics: LDL cholesterol = total cholesterol – HDL cholesterol + triglyceride/5. Insulin will be measured in the hospital’s endocrinology laboratory with the Elecsys 2010 and Modular Analytics E170/Cobas (F Hoffman–La Roche Ltd) by means of a kit that uses the immunoassay method of electrochemiluminescence. The cutoff points used for the evaluation of the serum levels of total cholesterol and fractions will follow the recommendation of the Brazilian Guideline for Dyslipidemias and Prevention of Atherosclerosis [35], and for fasting glycemia the parameter will be that recommended by the American Diabetes Association [36]. Undesirable levels of total cholesterol and LDL cholesterol values equal to or above 170 mg/dL and 110 mg/dL, respectively, and glucose above 100 mg/dL will be considered.

Insulin resistance will be assessed by means of the homeostatic model assessment–insulin resistance (HOMA-IR) index (HOMA-IR = fasting glycemia [mmol/L] × fasting insulinemia [μU/L]/22.5). Values >2.5 will be indicative of insulin resistance.

### Adherence to Intervention

Data regarding food consumption will be collected at each consultation by means of a 24-hour food recall using netbooks equipped with a multiple-pass method–based computer program for monitoring adolescent food consumption [37].

### Other Variables Investigated

At baseline, during the individual consultations, a questionnaire covering issues related to socioeconomic, demographic, and behavioral factors will be administered to the adults responsible for the children. Children will be asked about pubertal stage through the Tanner scale [38,39], sedentary activities (video games, television, and computer time), and physical activity using a validated questionnaire including 6 questions about frequency and duration of physical activities [40].

Family socioeconomic status will be evaluated using the Brazilian Economic Classification Criterion [41]. The number of sleeping hours will be ascertained from 3 questions based on 2 large epidemiological studies [42,43]: How many hours on average do you sleep on a normal night? Do you sleep fewer hours per night than you would like? How many hours would you like to sleep to feel like you have recovered? Sleep deprivation will be estimated from the difference between the usual hours of sleep and the hours of sleep the participant feels would facilitate recovery.

### Statistical Analysis

The rate of change of primary and secondary outcomes over time will be tested by intention-to-treat analysis based on mixed-effects models, which allow for consideration of incomplete follow-up data and take into account the correlation of repeated measures. Adequacy of the models will be checked graphically through the diagnosis of the residues [44]. The models will be adjusted for sexual maturation. For the analyses, SAS software version 9.4 (SAS Institute Inc) will be used.

## Results

This project was funded by the National Council for Scientific and Technological Development (CNPQ) in December 2017. The study protocol has undergone peer review by the funding body, which was not involved in the design of the study; collection, analysis, and interpretation of data; or in writing the manuscript.

Recruitment began in August 2018 and by September 2019, we had enrolled the 101 participants. In addition to the patients referred by the national system of regulation, recruitment was made by medical outpatient referral and external indication. This is an ongoing study. We expect the results to be published in November 2020.

## Discussion

The PAPPAS HUPE randomized clinical trial will be the first to provide data on the effectiveness of the recommendations of the Food Guide for the Brazilian Population on the treatment of obesity in children.

The possibility of incorporating NOVA food classification based on the degree of food processing for the treatment of obesity is unprecedented and timely, since this approach has been pointed out as a promising vehicle of nutritional education and also due to the high consumption of these products by Brazilian children.

The inclusion of parents in the activities is a strong point of the study. This approach can contribute to increased adherence to the protocol by leading to changes in family eating habits [45].

A possible limitation of this study is the difficulty in obtaining accurate measurements of food consumption according to the NOVA classification since it requires details about the degree of food processing. There is also a lack of consensus regarding the best data collection method because this is a recent technique. However, we will use the 24-hour recall, which is the standard collection method in food consumption assessment.

An intervention program combining quantity and quality approaches could be more efficient in the fight against obesity, especially in late childhood, when they acquire autonomy in relation to their diet. Moreover, we expect that the proposed nutritional plan would be able to promote weight loss without compromising growth and development. If effective, this proposal could guide the development of clinical protocols for primary care aimed at the treatment of obesity in children, an emergency challenge in the Brazilian and global public health agenda.

## Appendix

Multimedia Appendix 1 Previous peer-review report (Portuguese): CNPQ (Conselho Nacional de Desenvolvimento Científico e Tecnológico).

Multimedia Appendix 2 Previous peer-review report (English): CNPQ (National Council for Scientific and Technological Development).

Multimedia Appendix 3 SPIRIT checklist.

## Abbreviations

BF%: body fat percentage
BMI: body mass index
CNPQ: National Council for Scientific and Technological Development
HDL cholesterol: high-density lipoprotein cholesterol
HOMA-IR: homeostatic model assessment–insulin resistance
LDL cholesterol: low-density lipoprotein cholesterol
NC: neck circumference
PAPPAS HUPE: Parents and Professionals for Healthy Eating–Pedro Ernesto University Hospital
SISREG: Sistema Nacional de Regulação
WC: waist circumference
WHO: World Health Organization
WHR: waist-to-height ratio
