# Supplementary material for: Treatment of Childhood Obesity Based on Brazilian Dietary Guidelines Plus Energy Restriction (PAPPAS HUPE Study): Protocol for a Randomized Clinical Trial
Source: JMIR Res Protoc. 2020 Jun 8;9(6):e16170. doi: 10.2196/16170 (PMC7308900; doi:10.2196/16170)
Supplement: Multimedia Appendix 1 [file resprot_v9i6e16170_app1.pdf]

Resultado Final

1. **Identificação da Proposta**
2. **Número do Processo:** 408333/2017-0 **Solicitante:** Diana Barbosa Cunha
3. **Chamada:** PESQALIMENTNUTRI2017
4. **Título do Projeto:** Efetividade de uma estratégia para tratamento da obesidade infantil baseada no Guia Alimentar para a População Brasileira: um ensaio randomizado

1. Parecer de Deliberação Final
2.
   1. Nota Final
   2.

Nota	Ordem	Prioridade
9,45		

1. Resultado da Avaliação
2. Favorável

1. Justificativa:
2. Trata-se de estudo tipo "experimental ou ensaio clínico" para avaliar a efetividade da estratégia de adoção de um plano alimentar com medidas caseiras adequado às necessidades nutricionais das crianças e dos adolescentes baseada no Novo Guia Alimentar para a População Brasileira (grupo de intervenção), quando comparada às orientações egrais deste mesmo guia (grupo controle). Nesse caso, considerando que tanto o grupo de intervenção como o grupo controle receberá orientação nutricional baseada no Novo Guia Alimentar para a População Brasileira, a intervenção que está sendo avaliada é "o plano alimentar". Não fica claro se alguma comparação do tipo antes-depois será formalmente feita para a análise de efetividades das intervenções no grupo de intervenção e no grupo controle. A comparação explícita nos objetivos se refere à comparação do grupo controle com o grupo de intervenção. A proposta tem mérito, está plenamente justificada, a metodologia está clara (embora alguns aspectos mereçam maior detalhamento futuro), existe potencial para impactar nas ações direcionadas à esse público alvo, e o orçamento é compatível com os objetivos. A proponente é uma jovem doutora (2013), mas que já acumula certa produção científica, inclusive no tema e na metodologia proposta (ensaios clínicos), e em orientações de mestrado e doutorado. A equipe de pesquisadores tem membros com relevante experiência e produção científica na temática proposta.

1. Recursos
2.

Capital	Custeio	Bolsa	Valor Total
R$ 17.000,00	R$ 53.000,00	R$ 0,00	R$ 70.000,00

1. Data de Emissão
2. **Data de Emissão do Parecer:** 01/12/2017

1. Parecer de Deliberação final antes do período recursal
2.
   1. Critério: Mérito, originalidade e relevância do projeto para o desenvolvimento científico, tecnológico e a inovação do País.
   2. **Peso:** 3.0 **Nota:** 9.0

1. Critério: Pertinência e adequação da metodologia proposta
2. **Peso:** 2.0 **Nota:** 10.0

1. Critério: Experiência prévia do Coordenador da rede na área do projeto, considerando sua produção científica ou tecnológica relevante, nos últimos cinco anos.
2. **Peso:** 2.0 **Nota:** 9.0

1. Critério: Coerência e adequação entre a capacitação, perfil profissional eexperiência da equipe do projeto em relação aos objetivos, atividades e metas propostos.
2. **Peso:** 1.0 **Nota:** 10.0

1. Critério: Adequação e compatibilidade do orçamento aos objetivos, atividades e metas propostos.
2. **Peso:** 1.0 **Nota:** 10.0

1. Critério: Adequação do cronograma de entrega dos produtos esperados como resultado do projeto:
2. **Peso:** 1.0 **Nota:** 10.0

1. Critério: Contribuição da proposta para o uso do conhecimento e sua aplicação para a população em geral:
2. **Peso:** 1.0 **Nota:** 9.0

1. Nota Final
2.

Nota	Ordem	Prioridade

1. Resultado da Avaliação
2. Favorável

1. Justificativa:
2. Trata-se de estudo tipo "experimental ou ensaio clínico" para avaliar a efetividade da estratégia de adoção de um plano alimentar com medidas caseiras adequado às necessidades nutricionais das crianças e dos adolescentes baseada no Novo Guia Alimentar para a População Brasileira (grupo de intervenção), quando comparada às orientações egrais deste mesmo guia (grupo controle). Nesse caso, considerando que tanto o grupo de intervenção como o grupo controle receberá orientação nutricional baseada no Novo Guia Alimentar para a População Brasileira, a intervenção que está sendo avaliada é "o plano alimentar". Não fica claro se alguma comparação do tipo antes-depois será formalmente feita para a análise de efetividades das intervenções no grupo de intervenção e no grupo controle. A comparação explícita nos objetivos se refere à comparação do grupo controle com o grupo de intervenção. A proposta tem mérito, está plenamente justificada, a metodologia está clara (embora alguns aspectos mereçam maior detalhamento futuro), existe potencial para impactar nas ações direcionadas à esse público alvo, e o orçamento é compatível com os objetivos. A proponente é uma jovem doutora (2013), mas que já acumula certa produção científica, inclusive no tema e na metodologia proposta (ensaios clínicos), e em orientações de mestrado e doutorado. A equipe de pesquisadores tem membros com relevante experiência e produção científica na temática proposta.

1. Recursos
2.

Capital	Custeio	Bolsa	Valor Total
R$ 17.000,00	R$ 53.000,00	R$ 0,00	R$ 70.000,00

1. Data de Emissão
2. **Data de Emissão do Parecer:** 09/11/2017

1. Parecer de Recomendação
2.
    1. Critério: Mérito, originalidade e relevância do projeto para o desenvolvimento científico, tecnológico e a inovação do País.
    2. **Peso:** 3.0 **Nota:** 9.0

    1. Critério: Pertinência e adequação da metodologia proposta
    2. **Peso:** 2.0 **Nota:** 10.0

    1. Critério: Experiência prévia do Coordenador da rede na área do projeto, considerando sua produção científica ou tecnológica relevante, nos últimos cinco anos.
    2. **Peso:** 2.0 **Nota:** 9.0

    1. Critério: Coerência e adequação entre a capacitação, perfil profissional eexperiência da equipe do projeto em relação aos objetivos, atividades e metas propostos.
    2. **Peso:** 1.0 **Nota:** 10.0

    1. Critério: Adequação e compatibilidade do orçamento aos objetivos, atividades e metas propostos.
    2. **Peso:** 1.0 **Nota:** 10.0

    1. Critério: Adequação do cronograma de entrega dos produtos esperados como resultado do projeto:
    2. **Peso:** 1.0 **Nota:** 10.0

    1. Critério: Contribuição da proposta para o uso do conhecimento e sua aplicação para a população em geral:
    2. **Peso:** 1.0 **Nota:** 9.0

    1. Nota Final
    2.

Nota	Ordem	Prioridade

    1. Resultado da Avaliação
    2. Recomendada

    1. Justificativa:
    2. Trata-se de estudo tipo "experimental ou ensaio clínico" para avaliar a efetividade da estratégia de adoção de um plano alimentar com medidas caseiras adequado às necessidades nutricionais das crianças e dos adolescentes baseada no Novo Guia Alimentar para a População Brasileira (grupo de intervenção), quando comparada às orientações egrais deste mesmo guia (grupo controle). Nesse caso, considerando que tanto o grupo de intervenção como o grupo controle receberá orientação nutricional baseada no Novo Guia Alimentar para a População Brasileira, a intervenção que está sendo avaliada é "o plano alimentar". Não fica claro se alguma comparação do tipo antes-depois será formalmente feita para a análise de efetividades das intervenções no grupo de intervenção e no grupo controle. A comparação explícita nos objetivos se refere à comparação do grupo controle com o grupo de intervenção. A proposta tem mérito, está plenamente justificada, a metodologia está clara (embora alguns aspectos mereçam maior detalhamento futuro), existe potencial para impactar nas ações direcionadas à esse público alvo, e o orçamento é compatível com os objetivos. A proponente é uma jovem doutora (2013), mas que já acumula certa produção científica, inclusive no tema e na metodologia proposta (ensaios clínicos), e em orientações de mestrado e doutorado. A equipe de pesquisadores tem membros com relevante experiência e produção científica na temática proposta.

    1. Recursos

2.

Capital	Custeio	Bolsa	Valor Total
R$ 17.000,00	R$ 53.000,00	R$ 0,00	R$ 70.000,00

1. Data de Emissão
2. **Data de Emissão do Parecer:** 06/11/2017

1. Parecer de Pré-seleção
2.
   1. Critério: O proponente possui título de doutor?
   2. **SIM**

   1. Critério: O proponente possui vínculo formal com a instituição de execução do projeto?
   2. **SIM**

   1. Critério: A instituição de execução do projeto enquadra-se em uma das categorias definidas no subitem 3.3 da Chamada?
   2. **SIM**

   1. Critério: A proposta apresenta itens não financiáveis? Em caso afirmativo, descreva, no campo ?Comentários?, os itens não financiáveis com os respectivos valores.
   2. **SIM**

   1. Nota Final
   2. **Nota** **Ordem** **Prioridade**

   1. Resultado da Avaliação
   2. Enquadrado

   1. Justificativa:
   2. A proposta apresenta itens não financiáveis:"Serviço de tradução" e "Taxa de publicação de manuscrito". Ao Comitê Julgador para pronunciamento.

   1. Data de Emissão
   2. **Data de Emissão do Parecer:** 25/09/2017

1. Parecer de Ad Hoc
2.
   1. Critério: A proposta foi apresentada na forma de projeto de pesquisa, conforme recomendado no item 6.6 da Chamada? Se não, especifique, no campo ?Comentários?, o que faltou.
   2. **SIM**

   1. Critério: A proposta se adequa ao Tema/Linha de Pesquisa em que foi submetida?
   2. **SIM**

   1. Critério: Mérito, originalidade e relevância do projeto para o desenvolvimento científico, tecnológico e de inovação do País.
   2. **Excelente**

   1. Critério: Adequação da metodologia proposta.

2. **Bom**

1. Critério: Experiência prévia do Coordenador na área do projeto de pesquisa, considerando sua produção científica ou tecnológica relevante, nos últimos cinco anos, com base no CV Lattes.
2. **Excelente**

1. Critério: Coerência e adequação entre a capacitação e a experiência da equipe do projeto aos objetivos, atividades e metas propostos.
2. **Bom**

1. Critério: Adequação do orçamento aos objetivos, atividades e metas propostos.
2. **Excelente**

1. Critério: Adequação do cronograma de entrega dos produtos esperados como resultado do projeto:
2. **Excelente**

1. Critério: Contribuição da proposta para o uso do conhecimento e sua aplicação para a população em geral:
2. **Excelente**

1. Nota Final
2. **Nota** **Ordem** **Prioridade**

1. Resultado da Avaliação
2. Excelente

1. Justificativa:
2. O projeto tem o mérito de testar a efetividade da utilização das recomendações do Guia Alimentar para a População Brasileira no tratamento da obesidade em crianças e adolescentes. Também propõe a criação de um protocolo para tratamento da obesidade baseado no Guia Alimentar para a População Brasileira que poderá subsidiar a organização da Rede de Atenção à Saúde no Sistema Único de Saúde no enfrentamento da obesidade crescente em crianças e adolescentes.

1. Data de Emissão
2. **Data de Emissão do Parecer:** 13/09/2017

Voltar
