# Supplementary material for: Treatment of Childhood Obesity Based on Brazilian Dietary Guidelines Plus Energy Restriction (PAPPAS HUPE Study): Protocol for a Randomized Clinical Trial
Source: JMIR Res Protoc. 2020 Jun 8;9(6):e16170. doi: 10.2196/16170 (PMC7308900; doi:10.2196/16170)
Supplement: Multimedia Appendix 2 [file resprot_v9i6e16170_app2.pdf]

Final result
1. **Identification of the Proposal**
2. **Process Number:** 408333 / 2017-0 **Applicant:** Diana Barbosa Cunha
3. **Call:** PESQALIMENTNUTRI2017
4. **Project Title:** Effectiveness of a strategy for treating childhood obesity based on in the Food Guide for the Brazilian Population: a randomized trial

1. Final Deliberation Opinion
1. Final Note

Note
9.45

1. Evaluation Result
2. Favorable
1. Justification:
2. This is an "experimental or clinical trial" type study to assess the effectiveness of strategy of adopting a food plan with homemade measures appropriate to the nutritional needs of children and adolescents based on the New Guide Food for the Brazilian Population (intervention group), when compared to the general guidelines of this same guide (control group). In this case, considering that both the intervention group and the control group will receive nutritional guidance based on the New Food Guide for the Brazilian Population, the intervention that is being evaluated is "the eating plan". It is not clear whether any comparisons of the type before-after will be formally done to analyze the effectiveness of interventions in the intervention group and the control group. The explicit comparison in the objectives is refers to the comparison of the control group with the intervention group. The proposal has merit, is fully justified, the methodology is clear (although some aspects deserve greater detail in the future), there is potential to impact on the actions targeted to this target audience, and the budget is compatible with the objectives. The proponent is a young doctor (2013), but who already accumulates a certain production scientific research, including the proposed theme and methodology (clinical trials), and master's and doctorate guidelines. The research team has members with relevant experience and scientific production on the proposed theme.

1. Resources
2.

capital
Costing
handbag
Amount
R $ 17,000.00
R $ 53,000.00
R $ 0.00
R $ 70,000.00

1. Date of Issue
2. **Date of Issuance of the Opinion:** 12/01/2017
1. Final Deliberation Opinion before the appeal period
2.
1. Criterion: Merit, originality and relevance of the project for development scientific, technological and the country's innovation.
2. **Weight:** 3.0 **Note:** 9.0
1. Criterion: Relevance and adequacy of the proposed methodology
2. **Weight:** 2.0 **Note:** 10.0

1. Criterion: Previous experience of the network coordinator in the project area, considering its relevant scientific or technological production, in the last five years.
2. **Weight:** 2.0 **Note:** 9.0
1. Criterion: Coherence and adequacy between training, professional profile and experience
project team in relation to the proposed objectives, activities and goals.
2. **Weight:** 1.0 **Note:** 10.0
1. Criterion: Adequacy and compatibility of the budget to the objectives, activities and proposed goals.
2. **Weight:** 1.0 **Note:** 10.0
1. Criterion: Adequacy of the delivery schedule for the expected products as result of the project:
2. **Weight:** 1.0 **Note:** 10.0
1. Criterion: Contribution of the proposal to the use of knowledge and its application for the general population:
2. **Weight:** 1.0 **Note:** 9.0
1. Final Note
2.

Note
Order
Priority

1. Evaluation Result
2. Favorable
1. Justification:
2. This is an "experimental or clinical trial" type study to assess the effectiveness of strategy of adopting a food plan with homemade measures appropriate to the nutritional needs of children and adolescents based on the New Guide Food for the Brazilian Population (intervention group), when compared to the general guidelines of this same guide (control group). In this case, considering that both the intervention group and the control group will receive nutritional guidance based on the New Food Guide for the Brazilian Population, the intervention that is being evaluated is "the eating plan". It is not clear whether any comparisons of the type before-after will be formally done to analyze the effectiveness of interventions in the intervention group and the control group. The explicit comparison in the objectives is refers to the comparison of the control group with the intervention group. The proposal has merit, is fully justified, the methodology is clear (although some aspects deserve greater detail in the future), there is potential to impact on the actions targeted to this target audience, and the budget is compatible with the objectives. THE proponent is a young doctor (2013), but who already accumulates a certain production scientific research, including the proposed theme and methodology (clinical trials), and master's and doctorate guidelines. The research team has members with relevant experience and scientific production on the proposed theme.
1. Resources
2.

capital
Costing
handbag
Amount
R $ 17,000.00
R $ 53,000.00
R $ 0.00
R $ 70,000.00

1. Date of Issue
2. **Date of Issuance of the Opinion:** 11/09/2017

---

1. Opinion of Recommendation
2.
1. Criterion: Merit, originality and relevance of the project for development scientific, technological and the country's innovation.
2. **Weight:** 3.0 **Note:** 9.0
1. Criterion: Relevance and adequacy of the proposed methodology
2. **Weight:** 2.0 **Note:** 10.0
1. Criterion: Previous experience of the network coordinator in the project area, considering its relevant scientific or technological production, in the last five years.
2. **Weight:** 2.0 **Note:** 9.0
1. Criterion: Coherence and adequacy between training, professional profile and experience
project team in relation to the proposed objectives, activities and goals.
2. **Weight:** 1.0 **Note:** 10.0
1. Criterion: Adequacy and compatibility of the budget to the objectives, activities and proposed goals.
2. **Weight:** 1.0 **Note:** 10.0
1. Criterion: Adequacy of the delivery schedule for the expected products as result of the project:
2. **Weight:** 1.0 **Note:** 10.0
1. Criterion: Contribution of the proposal to the use of knowledge and its application for the general population:
2. **Weight:** 1.0 **Note:** 9.0
1. Final Note
2.

Note
Order
Priority

1. Evaluation Result
2. Recommended
1. Justification:
2. This is an "experimental or clinical trial" type study to assess the effectiveness of strategy of adopting a food plan with homemade measures appropriate to the nutritional needs of children and adolescents based on the New Guide
Food for the Brazilian Population (intervention group), when compared to the general guidelines of this same guide (control group). In this case, considering that both the intervention group and the control group will receive nutritional guidance based on the New Food Guide for the Brazilian Population, the intervention that is being evaluated is "the eating plan". It is not clear whether any comparisons of the type before-after will be formally done to analyze the effectiveness of interventions in the intervention group and the control group. The explicit comparison in the objectives is refers to the comparison of the control group with the intervention group. The proposal hasmerit, is fully justified, the methodology is clear (although some aspects deserve greater detail in the future), there is potential to impact on the actions

targeted to this target audience, and the budget is compatible with the objectives. THE
proponent is a young doctor (2013), but who already accumulates a certain production
scientific research, including the proposed theme and methodology (clinical trials), and
master's and doctorate guidelines. The research team has members with
relevant experience and scientific production on the proposed theme.
1. Resources

2.
capital
Costing
handbag
Amount
R $ 17,000.00
R $ 53,000.00
R $ 0.00
R $ 70,000.00
1. Date of Issue
2. **Date of Issue of the Opinion:** 11/06/2017
1. Pre-selection opinion
2.
1. Criterion: Does the applicant have a doctorate?
2. YES
1. Criterion: The tenderer has a formal link with the institution executing the project?
2. YES
1. Criterion: The institution implementing the project falls into one of the categories defined in sub-item 3.3 of the Call?
2. YES
1. Criterion: Does the proposal have non-financing items? If so, please describe, in the field? Comments ?, the non-financeable items with the respective values.
2. YES
1. Final Note
2.
Note
Order
Priority
1. Evaluation Result
2. Framed
1. Justification:
2. The proposal presents non-financeable items: "Translation service" and "Translation fee
publication of manuscript ". To the Judging Committee for pronouncement.
1. Date of Issue
2. **Date of Issue of the Opinion:** 09/25/2017
1. Ad Hoc Opinion
2.
1. Criterion: The proposal was presented in the form of a research project, as recommended in item 6.6 of the Call? If not, specify in the field?
Comments ?, what was missing.
2. YES

1. Criterion: Does the proposal suit the Theme / Line of Research to which it was submitted?
2. **YES**
1. Criterion: Merit, originality and relevance of the project for development scientific, technological and innovation in the country.
2. **Excellent**
1. Criterion: Adequacy of the proposed methodology.

---

2. **Good**
1. Criterion: The Coordinator's previous experience in the research project area, considering its relevant scientific or technological production, in the last five years, based on CV Lattes.
2. **Excellent**
1. Criterion: Coherence and adequacy between the training and experience of the team project to the proposed objectives, activities and goals.
2. **Good**
1. Criterion: Adequacy of the budget to the proposed objectives, activities and goals.
2. **Excellent**
1. Criterion: Adequacy of the delivery schedule for the expected products as result of the project:
2. **Excellent**
1. Criterion: Contribution of the proposal to the use of knowledge and its application for the general population:
2. **Excellent**
1. Final Note
2.

Note
Order
Priority

1. Evaluation Result
2. Excellent
1. Justification:
2. The project has the merit of testing the effectiveness of using the recommendations of the
Food Guide for the Brazilian Population in the treatment of obesity in children and teenagers. It also proposes the creation of a protocol for the treatment of obesity based on the Food Guide for the Brazilian Population, which may subsidize the organization of the Health Care Network in the Unified Health System in coping with increasing obesity in children and adolescents.
1. Date of Issue
2. **Date of Issue of the Opinion:** 09/13/2017
